# Understanding the challenges of non-food industrial product contamination

**DOI:** 10.1093/femsle/fnaa010

**Published:** 2020-01-16

**Authors:** Edward Cunningham-Oakes, Rebecca Weiser, Tom Pointon, Eshwar Mahenthiralingam

**Affiliations:** 1 Microbiomes, Microbes and Informatics Group, Organisms and Environment Division, Cardiff School of Biosciences, Cardiff University, Park Place, Cardiff, UK, Wales CF10 3AX, UK; 2 Unilever Research and Development, Port Sunlight, Wirral, CH62 4ZD, UK

**Keywords:** contamination, non-food products, antimicrobial resistance, classification, identification

## Abstract

Preventing microbial contamination of non-food products is a major area of industrial microbiology where preservatives are used to stop microbial growth. However, microorganisms occasionally overcome product preservation, causing recalls and the implementation of multiple procedures to prevent further contamination. Correct reporting of microbial contamination in non-food industrial products is vital, especially if spoilage organisms are antimicrobial resistant and pose a health threat. Gram-negative bacteria such as *Pseudomonas, Burkholderia* and *Enterobacteriaceae* are frequently reported as non-food product contaminants, including species that overlap current antimicrobial resistance priorities. Historical analysis of recall databases highlighted that for greater than 15% of contamination incidents, the causative microbial agents are reported as unidentified. Here we review the current antimicrobial resistant bacterial species associated with non-food product contamination and evaluate recall reporting in Europe from 2005 to 2018. Our review shows that 49% of microbial contaminants are reported as unidentified despite frequent detection of antimicrobial resistant pathogens; in contrast, 98% of food-related microbial contaminants are classified. Recommendations to fill this microbial identification gap in non-food product recalls are made. Overall, reporting standards for microbial contamination in non-food products must be improved to enable surveillance and for understanding the risks associated with antimicrobial resistant microorganisms

## Teaser

Microbial contamination in non-food industrial products was reviewed and it is shown that while antimicrobial resistant Gram-negative species are the most common cause, nearly 50% of recorded recall incidents report the microorganisms present as unidentified.

Preventing microbial spoilage is a key issue for several industrial sectors including the manufacture and sale of non-food products such as home care products (cleaning products), personal care products (cosmetics and toiletries) and toys. Preservatives and antimicrobial formulations are used to prevent microbial growth in non-food products, but since manufacture of these products is not a sterile process, growth of certain resilient and intrinsically antimicrobial resistant (AMR) microorganisms may occur. Non-food product microbial contamination is a costly issue for industry and reporting of incidents associated with publicly recalled products is required. We review bacterial groups most commonly associated with non-food product contamination, focussing on *Pseudomonas aeruginosa*, *Burkholderia cepacia* complex and members of *Enterobacteriaceae*. Evaluation of current recall data for Europe as part of this exercise worryingly identified that half of all non-food microbiological contamination incidents reported the causative agent as unidentified. The issues behind the lack of microbial contaminant identification in the non-food product sector are discussed and recommendations to improve reporting are made.

The emergence of treatment refractory infections such as the notorious ESKAPE pathogens (*Enterococcus faecium, Staphylococcus aureus, Klebsiella pneumoniae, Acinetobacter baumannii, P. aeruginosa* and *Enterobacter spp*.) has been extensively highlighted, and these organisms contribute greatly to patient morbidity and mortality (Pendleton, Gorman and Gilmore 2013). Intrinsically resistant microorganisms are also problematic in manufacturing settings and can overcome antimicrobial preservatives to contaminate industrial products. *Pseudomonas* and multiple *Enterobacteriaceae* (eg. *Enterobacter* and *Klebsiella*) are globally recognised clinical AMR threats (Tacconelli *et al*. 2018a), but they and other intrinsically resistant bacteria such as *Burkholderia* may also be recovered as microbial contaminants of non-food products (Jimenez 2007; Sutton and Jimenez 2012; Vincze, Al Dahouk and Dieckmann 2019). Multiple Gram-negative bacteria have intrinsic resistance and the potential to acquire AMR elements (Pendleton, Gorman and Gilmore 2013), but what is their role in non-sterile product contamination and do they constitute potential public health risks? The identification and tracking of antimicrobial resistant contaminants in non-food consumer products is far less stringent than in clinical settings and the food product industry, making a thorough risk assessment difficult. In this review we also expand on key non-food product contaminating AMR bacteria and issues in relation to their identification and reporting.

## MICROBIAL ISSUES ASSOCIATED WITH THE MANUFACTURE OF NON-STERILE INDUSTRIAL PRODUCTS

Non-food consumer products encompass an extensive range of items including cosmetics, personal hygiene and home care products, chemical products such as tattoo ink and children's toys such as soap bubbles, plasticine and paints (European Commission 2018). These products are manufactured, sold and used world-wide and are not required to be sterile, but must be safe for use (Orus and Leranoz 2005). Antimicrobial preservatives are incorporated into products to maintain their quality, extend shelf life and protect the consumer (Neza and Centini 2016). If a product is inadequately preserved, microbial contamination may occur which can lead to product spoilage and recall, financial or reputational damage to the manufacturer, and more seriously, poses a risk to human health (Orth *et al*. 2006). Alarmingly, the presence of opportunistic pathogens in non-food products has been linked with infections in vulnerable consumers (Lundov *et al*. 2009). Whilst incidents are most likely to be reported in hospital settings, contaminated products do reach the market place, as has been shown by product recall data and surveys of off-the-shelf products (Lundov *et al*. 2009). Despite being a major area of routine global microbiology, the risks stemming from contaminated non-food products are poorly characterised and literature in this area is limited.

## RECALL INFORMATION IS USEFUL FOR SURVEILLANCE OF MICROBIAL CONTAMINATION

As there is no requirement for industry to publish information concerning products which have failed quality control or contamination incidents, systematic literature in this area is limited. Product recall data, however, is collated by the US Food and Drug Administration (FDA) (Food and Drug Administration 2017) and the European Commission (European Commission 2018), and made publicly available. Reports based on FDA data for personal care and non-sterile pharmaceutical products (Jimenez 2007; Sutton and Jimenez 2012) and Safety Gate (previously known as RAPEX: the rapid alert system for non-food consumer products) (European Commission 2018) in the EU (Wong *et al*. 2000; Lundov *et al*. 2009), indicate the range of microorganisms found as contaminants. A report published in 2007 (Jimenez 2007) surveying the FDA database from 1995 to 2006, documented that Gram-negative bacteria accounted for 60% of product recalls, with Gram-positive bacteria and fungal species accounting for approximately 4% and 23%, respectively. Microorganisms recorded as unidentified comprised 22% of the recall cases evaluated at the time (Jimenez 2007: 383–99). A subsequent update examined FDA recalls from 2004 to 2011 (Sutton and Jimenez 2012), and identified a similar proportion of Gram-negative bacteria (50%), Gram-positive bacteria (10%) and fungal (19%) contaminants, but showed limited improvement in the number of recall incidents reported due to unidentified microorganisms (15%). Overall, these pioneering surveillance reports clearly showed that Gram-negative bacterial species caused over half of the incidents which resulted in recall of non-sterile industrial products, and also demonstrated that a significant proportion of the non-food product contamination microorganisms were left unidentified.

Gram-negative bacterial species stand out as the most common contaminants of non-sterile industrial products. Historically, *P. aeruginosa* and *B. cepacia* complex bacteria, have been recognised as problematic, objectionable industrial contaminants, identified in a high proportion of recall incidents. *P. aeruginosa* accounted for 14% and 6% of recall incidents respectively for 1995 to 2006 and 2004 to 2011 FDA database surveys (Jimenez 2007; Sutton and Jimenez 2012); over the same time periods *B. cepacia* complex bacteria were identified in 22% and 25% of incident surveys (Jimenez 2007; Sutton and Jimenez 2012). *P. aeruginosa* was the most common contaminant reported in recalled cosmetic products in a short survey (2005 to 2008) of the RAPEX Safety Gate database (Lundov and Zachariae 2008). *Enterobacteriaceae* are also prominent contaminants with significant numbers of *Enterobacter* species identified within recalled products in the United States (Jimenez 2007; Sutton and Jimenez 2012). A recent update examining contaminant notifications in the Safety Gate database between 2005 and 2017 also found that *P. aeruginosa*, other *Pseudomonas* species and *Enterobacteriaceae* ranked as the top three identified microorganisms encountered in contaminated toys, cosmetics and chemical products (Vincze, Al Dahouk and Dieckmann 2019). However, Vincze *et al*. (Vincze, Al Dahouk and Dieckmann 2019) also determined that the main microbial hazard was an unacceptably high total count of unidentified aerobic microorganisms; 218 out of 240 products were recalled for this reason, only half of which had additional microbial identification information.

## THE IDENTIFICATION GAP: A LACK OF ORGANISM CLASSIFICATION FOR NON-FOOD PRODUCT RECALL INCIDENTS

The previously mentioned meta-analyses of both FDA and EU product recall databases have determined that unidentified microorganisms are associated with a large proportion of product recalls. To update these past analyses (Jimenez 2007; Lundov and Zachariae 2008; Sutton and Jimenez 2012), investigate recall reporting in different industrial sectors, and determine the extent of AMR microorganisms within contamination incidents, we conducted a meta-analysis of both food and non-food product recalls between 2005 and 2018 in the EU. To do this we used two publicly available EU databases, (i) the European Commission Rapid Alert System for Food and Feed (RASFF) (European Commission 2019) to search food product recalls, and (ii) Safety Gate (European Commission 2018) to search non-food product recalls. These databases record a variety of parameters for each recall including the nature and severity of risk, product type, country of origin, notifying country and overall outcomes. In relation to contaminated foods, searches of RASFF database were performed for: (i) product type ‘Food’, and (ii) hazard type ‘Pathogenic microorganisms’ or ‘Non-pathogenic microorganisms’ and each incident recorded within a spreadsheet. An analogous search of the Safety Gate database for all product categories and the risk type ‘Microbiological’ was also performed. The classification of each reported microorganism was recorded to the family, genus or species level. Organisms not classified to at least family level or grouped together under collective descriptions such as ‘coliforms’, ‘moulds’ or ‘fungus’, were designated ‘unidentified’. If multiple organisms were found in a product recall, each organism was tallied separately.

**Figure 1.**
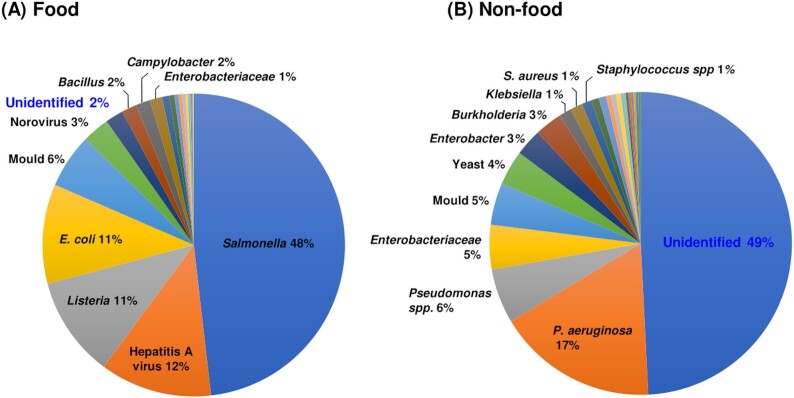
Lack of microbial species identification in non-food product recalls compared to food products. The diversity of microorganisms responsible for product recalls in the European databases was analysed for food and non-food products between 2005 and 2018. The proportion of organism types detected for the 7577 food product and 378 non-food product isolations made in recall incidents is shown in panels A and B, respectively. Each labelled segment highlights where organisms represented ≥1% of the total number of recall reports in the period. Unidentified organisms in each database are highlighted in the blue font.

For food products over the 2005 to 2018 period, a total of 7577 microorganisms were reported in 1016 recalls, while in contrast a total of 378 microorganisms were reported for 254 non-food product incidents over the same time period. To probe the extent of useful epidemiological information within the RASFF and Safety Gate databases, information related to the identity of the contaminating organism for each product recall was examined. Proportional analysis of the microbial groups encountered in food and non-food product contamination revealed major differences in the type of microorganisms reported in recalls (Fig. 1). The top five microorganisms encountered in food product recalls in rank order were *Salmonella*, Hepatitis A virus*, Listeria, Escherichia coli* and moulds (Fig. 1A). Given the major importance of *Salmonella* as a food-bourne pathogen it was not surprising that this Gram-negative genus was identified in 48% of food product recalls. An additional key feature of the recall information in the food product database was the accuracy of identification, with 98% of organisms being identified and only 2% being reported as unidentified (Fig. 1A).

**Figure 2.**
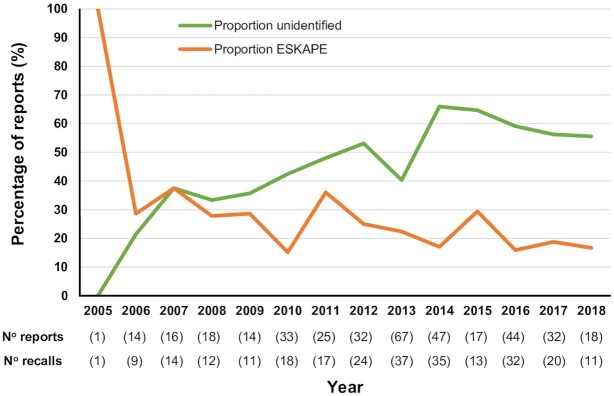
The proportion of unclassified microbial contaminants recorded for non-food consumer products is increasing. The proportion of reports of microbial contaminants in EU Safety Gate non-food product recalls from 2005 to 2018 associated with unidentified microorganisms (green line) and ESKAPE pathogens (*E. faecium*, *S. aureus*, *K. pneumoniae*, *A. baumannii*, *P. aeruginosa* and *Enterobacter* spp.; orange line). The total number of reports of microorganisms and corresponding number of product recalls is given below the year in brackets.

In striking contrast to the food related recalls, 49% of microorganisms associated with non-food product recalls were reported as unidentified (Fig. 1B). After this, the top ten most common microorganisms in non-food product recalls, from highest to lowest prevalence, were *P. aeruginosa*, *Pseudomonas* species, *Enterobacteriaceae*, Moulds, Yeasts, *Enterobacter*, *Burkholderia, Klebsiella*, *S. aureus* and *Staphylococcus* species (Fig. 1B). Overall, the intrinsically antimicrobial resistant Gram-negative bacteria *Pseudomonas*, *Enterobacteriaceae*, *Enterobacter*, *Burkholderia, Klebsiella* and *Achromobacter*, were associated with 30% of non-food product recalls (Fig. 1B). In addition, the ESKAPE pathogen group of bacteria (Pendleton, Gorman and Gilmore 2013) accounted for 23% of all non-food recalls (data not shown). If the same proportions of antimicrobial resistant ESKAPE species are mirrored in the 49% of recall microorganisms recorded as unidentified, then the non-food product manufacturing industry is considerably under-reporting potential hazards in terms of global health risk and AMR. Given the recent significant improvements in methods for accurate microbial identification (Maiden *et al*. 2013; van Belkum *et al*. 2013) it would be expected that more contamination incidents would be accurately reported over time. Between 2005 to 2018, the number of non-food product recalls varied between 1 and 37 incidents per year, with the number of reports of microorganisms varying between 1 and 67 (Fig. 2). Proportionally, the lack of identification in reporting for non-food product recall microorganisms increased from 2010 onwards to a high in 2014 where 66% of incidents were reported as unidentified (Fig. 2). Overall, to the current time there remains a high proportion of unidentified contaminants (>55%) in non-food product recalls (Fig. 2).

## NON-FOOD PRODUCT TYPES MOST AFFECTED BY MICROBIAL CONTAMINATION

**Figure 3.**
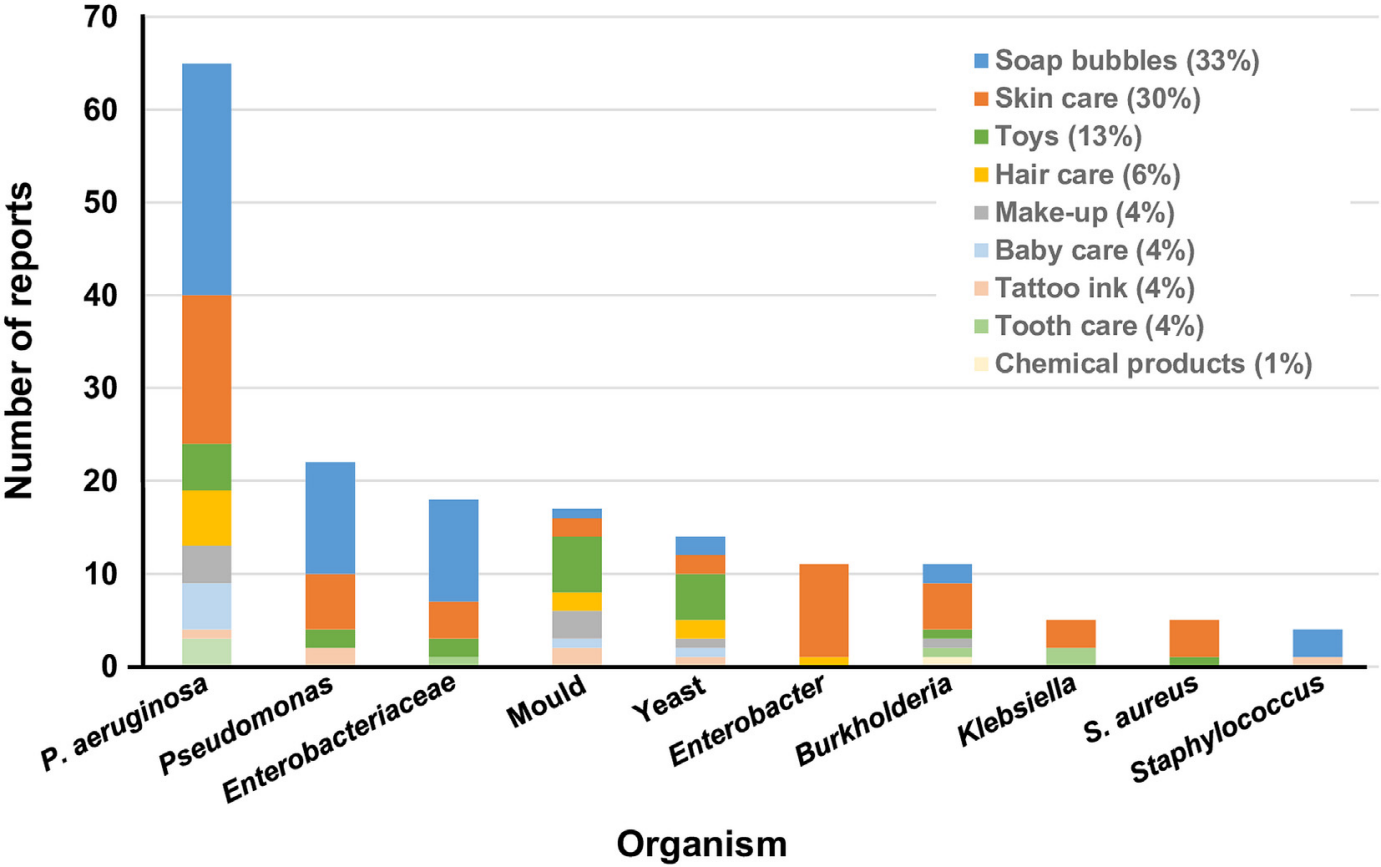
The microbial groups and species most commonly reported in non-food product types. The top 10 microorganisms identified from recalled non-food product types within the Safety Gate database are shown. The data derives from 172 reports of microbial contamination from 138 product recalls between 2005 to 2018 where an organism identification was provided for the non-food product type. The percentage of reports belonging to each product category is shown in the colour key at the top right of the figure.

The top 10 identified microorganisms in product recalls between 2005 and 2018 collectively represented 172 reports of microbial contamination, which were linked to 138 product recalls belonging to nine different product categories (Fig. 3; soap bubble products, face/hand/feet/body care, toys, hair care, make-up, baby care, tattoo ink, tooth care and chemical products). Soap bubbles (33% of reports) and face/hand/feet/body care (skin care) products (30% of reports) were the most commonly reported contaminated products (Fig. 3). In rank order below this, contamination of toys was linked to 13% of the microbial reports, while hair care, make-up, baby-care, tattoo ink, tooth care and undefined chemical products each accounted 6% or less (Fig. 3). *P. aeruginosa* showed the most widespread distribution being identified in recalls of eight of the nine product types (it was not found in chemical products), as well as being most commonly found in soap bubble and skin care products (Fig. 3). Other *Pseudomonas* species were encountered in four of the nine products types, and in correlation with *P. aeruginosa*, they affected soap bubble and skin care products most commonly. *Enterobacteriaceae* also showed a propensity to contaminate soap bubble and skin care products, and 10 of the 11 ‘*Enterobacter*’ species-linked reports occurred specifically in skin care products (Fig. 3). *Burkholderia, Klebsiella* and *S. aureus* were most commonly encountered in recalled skin care products (Fig. 3). Overall, *P. aeruginosa* represented the most substantive risk of contamination across multiple non-food product types (Fig. 3).

## FREQUENT CONTAMINANTS OF NON-FOOD PRODUCTS: *P. AERUGINOSA*

*P. aeruginosa* is an extremely versatile microorganism with the ability to survive in diverse habitats including soil, water, plant and animal tissues, community and hospital environments. In particular, its presence within water via natural, tap and potable sources is a recognised problem for manufacturing industries reliant on water as a major raw material (Jimenez 2007). *P. aeruginosa* is a rapidly growing microorganism and is straightforward to culture with a range of positive or negative selective media (Weiser *et al*. 2014). Standard industrial practices for microbiology and quality control are effective for recovering it as a contaminant from a range of products and industrial sources (White *et al*. 2011; Weiser *et al*. 2014). Accurate identification of *P. aeruginosa* can be achieved using multiple methods including biochemical profiling or matrix-assisted laser desorption-ionisation time of flight mass spectroscopy (MALDI TOF MS) (van Belkum *et al*. 2013), 16S rRNA or *oprL* gene (De Vos *et al*. 1997) sequence analysis and international databases such as the public multilocus sequencing typing (MLST) resource (https://pubmlst.org/paeruginosa/) (Curran *et al*. 2004) can enable placement of strains in the context of their wider epidemiological prevalence.

Contamination of taps, surfaces, instruments and patients has been associated with multiple incidents of nosocomially acquired *P. aeruginosa* infections. The presence of the pathogen in home and personal care products has also initiated infections in hospital settings. A historical case associated with a contaminated hand lotion used by healthcare workers in a neonatal intensive care unit inadvertently led to vulnerable infants being infected (Becks and Lorenzoni 1995). Interestingly, the *Enterobacteriaceae* species *Enterobacter agglomerans* was also cultured from the contaminated hand lotion bottles, but did not cause infection in the infants (Becks and Lorenzoni 1995). More recently an outbreak with extensively drug-resistant *P. aeruginosa* was associated with sharing of an aromatherapy oil among hospitalised patients in an intensive care unit (Mayr *et al*. 2017). Such localised incidents of contamination leading to infection can be normally be controlled by infection control, but outbreaks in wider community settings are problematic to resolve. A national *P. aeruginosa* outbreak in the UK occurred among individuals undergoing ear piercing, which continued for 3 months in 2016 before it was stopped by a source tracing enabled product recall (Evans *et al*. 2018). Using variable Number Tandem Repeat (VNTR) strain typing and cohort analysis, the outbreak was linked to the use of a cosmetic after-care solution which had become contaminated during production (Evans *et al*. 2018). Within the same industrial sector of beauty and cosmetic products, contaminated tattoo ink has also been implicated in multiple cases of *P. aeruginosa* infection (Hogsberg *et al*. 2013). Overall, *P. aeruginosa* in addition to being frequently described as ubiquitous bacterial species found in multiple niches, is consistently encountered in relation to incidents of non-food product contamination (Jimenez 2007; Sutton and Jimenez 2012).

## *B. CEPACIA* COMPLEX BACTERIA

*B. cepacia* complex bacteria are also historically linked with the ability to cause contamination in both clinical and non-food industrial settings (Torbeck *et al*. 2011). This group of Gram-negative bacteria has undergone multiple taxonomic revisions over the last 20 years with over 20 species defined within the *B. cepacia* complex (Depoorter *et al*. 2016). The historical prevalence of individual *B. cepacia* species in contamination is more difficult to assess since it was reported as ‘*Pseudomonas cepacia*’ up to 1997 prior to taxonomic reclassification (Vandamme *et al*. 1997), and even post 2010, it is still occasionally referred to as ‘*Burkholderia cepacia*’ in the published literature (Torbeck *et al*. 2011). Unlike *P. aeruginosa*, *B. cepacia* complex bacteria are not straightforward to identify, requiring selective media for their enrichment (Henry *et al*. 1999), and molecular tests such as *recA* gene sequencing (Mahenthiralingam *et al*. 2000) and MLST (Baldwin *et al*. 2005) for accurate identification. Surveillance and tracking of infections caused by *B. cepacia* complex bacteria has been extensive because of the problematic lung disease they cause in people with cystic fibrosis, and MLST resources for strain identification are comprehensive (Baldwin *et al*. 2005) (https://pubmlst.org/bcc/).

In the last 5 years, analysis of non-food product contamination caused by *B. cepacia* complex bacteria has been more detailed in relation to reporting taxonomic identity. Species associated with a variety of non-food product sources were accurately identified within a collection of 60 industrial isolates, and *Burkholderia lata, Burkholderia cenocepacia* and *Burkholderia vietnamiensis* were the top three species accounting for 25%, 18% and 13%, respectively, of the collection assessed (Rushton *et al*. 2013). In 2016, a strain of *Burkholderia stabilis* was shown to cause an outbreak associated with contaminated washing gloves in Switzerland using a comprehensive range of molecular and genomic tests (Sommerstein *et al*. 2017). Whole genome sequencing including whole genome MLST (Maiden *et al*. 2013) was carried out to identify that the *B. stabilis* isolates causing infections in multiple patients at different treatment centers were identical to those recovered from the contaminated gloves (Sommerstein *et al*. 2017). In the United States, a widespread outbreak associated with the laxative docusate occurred in 2016, and isolates causing the resulting infections in patients shown to be identical using a PCR genotyping method (Marquez *et al*. 2017). However further molecular analysis demonstrated that the docusate outbreak strain belonged to a potentially novel taxonomic group within the *B. cepacia* complex.

Although improvements in the level of resolution being applied to the investigation of *B. cepacia* complex contamination are clearly being made, studies may still fail to follow up on the resources obtained during the investigation of outbreak incidents. For example, while whole genome sequencing was applied to demonstrate that a clonal *B. cepacia* complex strain present in a contaminated octenidine mouthwash had caused an outbreak of clinical infections, further follow-up to identify the strain to a species level was not performed (Becker *et al*. 2018). The availability of whole genome sequence data offers multiple bioinformatic approaches to accurately assign species status (Bull *et al*. 2012) and excellent comparative resources for sequence data are available at the *B. cepacia* complex MLST site (https://pubmlst.org/bcc/). Using the deposited whole genome sequence of the octenidine contamination isolates (Becker *et al*. 2018), an MLST sequence type was derived, ST-881. This showed that the mouth wash and outbreak strain (Becker *et al*. 2018) was a member of the *B. cepacia* complex species *Burkholderia arboris*. Although, this retrospective analysis of recently deposited genome sequence data for a *B. cepacia* complex contaminant (Becker *et al*. 2018) was carried out using command-line bioinformatics, the same identification result of *B. arboris* was also obtained when the genome sequence data was uploaded on the web to the MLST database and a search for matching sequence loci initiated.

## ENTEROBACTERIACEAE

Multiple *Enterobacteriaceae* including *Klebsiella*, *Enterobacter* (European Commission 2018) and *Serratia* (Polilli *et al*. 2011) have been encountered as non-food product contaminants, and recent reports show certain species within this highly related group of Gram-negative bacteria have become quite problematic. In particular, *Enterobacter gergoviae* is a well-known contaminant of food and cosmetic products with high intrinsic preservative resistance (Periame, Pages and Davin-Regli 2014). Recent taxonomic reclassification of several *Enterobacter* species placed *E. gergoviae* isolated within a new genus *Pluralibacter* using multilocus sequence-based methods (Brady *et al*. 2013). A recall of 15 000 tubes of a best-selling skincare product because of high levels of *Pluralibacter (Enterobacter) gergoviae* was reported in the media in 2016, bringing public attention to the issue of non-food product contamination by this species. In addition, *P. gergoviae*, has been responsible for seven other major European recall incidents involving skin care products in the past ten years, as documented by Safety Gate (European Commission 2018).

The close taxonomic relationships between *Enterobacteriaceae* species and their propensity to cause infection and product contamination underpin the need for accurate and high resolution identification methods. Over the past two decades, such methods have been developed and applied predominantly in clinical and research settings, but also have application in non-food product contamination. Many genera within *Enterobacteriaceae* are polyphyletic by 16S rRNA gene sequence analysis alone and therefore expanded genotyping analyses have been required for taxonomic assignments (Brady *et al*. 2013). As previously mentioned, multilocus sequence-based methods were used to re-classify *Enterobacter* species as different genera (Brady *et al*. 2013) and a limited number of studies have applied similar techniques to contamination scenarios. An investigation of a contaminated batch of liquid hand soap identified a single strain of *Klebsiella oxytoca* was present in the product bottles and clearly illustrates that accurate species identification can be achieved in the non-food product sector (Dieckmann *et al*. 2016). In this study, a combination of rapid phenotypic methods (MALDI TOF MS and infrared spectroscopy) and genotypic methods (pulsed-field gel electrophoresis, MLST and whole genome sequencing) were used to accurately identify the contaminant to both the species and strain level. Whilst there are multiple media for culture and putative phenotypic identification of *Enterobacteriaceae* (Perry 2017) genotyping and whole genome sequencing have vastly increased accuracy and resolution. Resources such as the extensive genomic databases within Enterobase (https://enterobase.warwick.ac.uk) are available to facilitate detailed characterisation of strains from certain *Enterobacteriaceae* (Alikhan *et al*. 2018).

## THE ISSUES BEHIND A LACK OF IDENTIFICATION OF NON-FOOD PRODUCT CONTAMINANTS

The large discrepancy in the proportion of unidentified organisms reported in food and non-food product recall information (Figs 1 and 2) may reflect a number of issues with how contaminants are identified and validated for the hygienic integrity of products. The RASFF (European Commission 2019) and Safety Gate (European Commission 2018) databases do not publish microbial identification methods and there is likely to be considerable variability between reporting sites and resource limitations when routine testing only detects certain microorganisms. The perceived risk may also influence the extent of reporting, with more stringent quality control being applied to foods that will be ingested, compared to non-food products having more limited direct contact with consumers. The non-food global ISO 11 930 and European Pharmacopeia challenge testing methods require the absence of *E. coli*, *P. aeruginosa*, *S. aureus*, *Candida albicans* and *Aspergillus brasiliensis* (Vincze, Al Dahouk and Dieckmann 2019); but do not require evaluation against opportunistic pathogens *Enterobacter*, *Burkholderia* or *Klebsiella* species. From our meta-analysis (Fig. 1B) it is clear that *Enterobacter*, *Burkholderia* and *Klebsiella* are causing a growing number of non-food product recalls and should be integrated into challenge test methodologies to ensure products are adequately preserved for their intended purpose.

Current regulations require a mandatory absence from a representative sample (1 g or 1 mL) of the following microorganisms: for cosmetics, *P. aeruginosa*, *S. aureus* and *C. albicans* must be absent; and for toys, the latter organisms in addition to *E. coli* and *Salmonella* species must be absent (Vincze, Al Dahouk and Dieckmann 2019). Hence a significant number of incidents reported as containing unidentified microorganisms could be attributable to genera such as *Enterobacter*, *Burkholderia* and *Klebsiella* that are not listed as mandatory for reporting. Overall, in relation to the clinical and environmental research areas, industrial microbiology receives limited attention despite the global scale and daily usage of non-food products. It is apparent from the information recorded in the recall database that a range of antimicrobial resistant microorganisms and specific ESKAPE pathogens (Pendleton, Gorman and Gilmore 2013) may be encountered as non-food product contaminants (Fig. 1B). Since these organisms must survive in the presence of antimicrobials to contaminate preserved non-food products, they represent an additional ecological niche for investigation of AMR. Whilst biocide and preservative resistance is mediated by complex, multifactorial mechanisms and is often transient (Maillard 2007), stable adaptive changes resulting in preservative tolerance and cross-resistance to antibiotics have been observed for the opportunistic pathogens such as *P. aeruginosa* (Abdel Malek and Badran 2010: 588–92) and *B. cepacia* complex bacteria (Rushton *et al*. 2013). Isolation and characterisation of strains of industrial origin with increased AMR is not just important to understand resistance mechanisms but can also impact industrial practices directly. In a case study from 2003 (Ferrarese, Paglia and Ghirardini 2003), *P. aeruginosa* and *P. gergoviae* strains originating from an industrial plant were found to have increased resistance to cosmetic preservatives, in particular formaldehyde releasing agents. This led to the evaluation of disinfection procedures in the plant and change to a more efficacious decontamination regime.

Correct classification of bacterial infections is essential for accurate local and global microbiological surveillance. It has been deemed vital to improve surveillance to understand the health threat of AMR and ensure antibiotic stewardship programmes are successful (Tacconelli *et al*. 2018b). Multiple strategies are currently available to accurately identify microorganisms to genus or species level, including phenotypic (biochemical analysis or MALDI TOF MS) (van Belkum *et al*. 2013) and genotypic methods (16S rRNA gene sequencing, MLST or whole genome sequencing) (Maiden *et al*. 2013). Whole genome sequencing has revolutionised our understanding of bacteria, facilitating detailed epidemiological understanding of both hospital acquired infection and food-contamination incidents (Maiden *et al*. 2013). Currently, routine microbial identification in the non-food product industry is based on culture-dependent methods (European Commission 2016; Food and Drug Administration 2017) which have numerous limitations. The expense of molecular diagnostics and DNA sequencing is no longer prohibitive due to the rapid improvement in technology and curtailment in costs. It is therefore the opportune time for wider industrial sectors to take advantage of the accurate microbial identification afforded by DNA sequence-based and gold standard genomic methods (Maiden *et al*. 2013). All of these techniques, including genome sequencing are now employed regularly in clinical microbiology and food microbiology to identify and track priority pathogens.

## RECOMMENDATIONS TO FILL THE NON-FOOD PRODUCT MICROBIAL IDENTIFICATION GAP

If the trend towards reporting microorganisms as unidentified in the Safety Gate database is not corrected, researchers, manufacturers and the public will not have an accurate means to understand the risks posed by non-food product contaminating microorganisms. Methods to accurately identify microorganisms have improved considerably (van Belkum *et al*. 2013) and multiple commercial companies offer both MALDI TOF MS and gene/genome sequence-based analysis for relatively limited costs. The costs of microbial identification in relation to recall incidents is also small in relation to the overall costs of manufacturers having to deal with removing products, cleaning manufacturing facilities, or develop new formulations to prevent non-food product contamination. Further to the methods to identify *P. aeruginosa, B. cepacia* complex and *Enterobacteriaceae* discussed above, *Stapyloccocus* and fungal species were also among the top five non-food product contaminants (Fig. 1B). *Staphylococcus* species can be selectively cultured and identified on a range of growth media (Perry 2017). DNA sequence-based identification using the 16S rRNA and *rpoB* genes is also effective (Mellmann *et al*. 2006) and MLST databases are available for multiple pathogenic species (https://pubmlst.org/databases). Systematic identification of fungi is less advanced than bacteria but sequence analysis of the nuclear ribosomal DNA internal transcribed (ITS) is an accurate means to validate species groups (Raja *et al*. 2017).

In terms of improving recall and surveillance information, a primary recommendation would be that non-food product recall reports require microbial identification to be carried out to a minimum of the genus level for both bacteria and fungi. A secondary recommendation would be that for bacterial species which pose a significant health or AMR risk as opportunistic pathogens (e.g. *P. aeruginosa*, the *Enterobacteriaceae*, *B. cepacia* complex and *Staphylococcus spp*.), identification to the species level should also be reported. In addition, identification should be checked against the current taxonomy of each species, for example by comparison to the List of Prokaryotic names with Standing in Nomenclature (www.bacterio.net) (Parte 2018), to enable fine-grain surveillance and accurate comparative analysis of microbial contamination incidents. Harmonisation of detection methods and minimum identification criteria across EU laboratories is necessary to improve the consistency of reporting, bringing it closer to the level of reporting seen in food products (Vincze, Al Dahouk and Dieckmann 2019).

If manufacturers and regulatory bodies apply whole genome sequencing to the characterization of contaminant and outbreak strains, then they have all the information required for accurate species classification (Bull *et al*. 2012). Such analysis can also future-proof our understanding by archiving sequence data within nucleotide sequence databases to enable retrospective and comparative analysis of contamination incidents. In addition, since the non-food product and cosmetics industry landscape is changing, methods for surveillance and recall recording need to be updated to facilitate the identification of contamination trends. The rise of new market trends such as natural preservative ingredients in non-food products such as cosmetics (Papageorgiou *et al*. 2010) may result in a shift in the diversity of microorganisms encountered as contaminants. Improved vigilance of non-food microbial contamination is also key to ensure that testing methodologies remain relevant to the products they govern. For example, the non-food global ISO 11 930 and European Pharmacopeia testing methods do not currently include the prevalent contaminating bacteria *Enterobacter*, *Burkholderia* or *Klebsiella* (Fig. 1), and only recommend using *E. coli*, *P. aeruginosa*, *S. aureus*, *C. albicans* and *A. brasiliensis* (Siegert 2013). The inclusion of *Burkholderia* as mandatory for reporting has already been discussed (Torbeck *et al*. 2011), and as other important opportunistic pathogens, *Enterobacter* and *Klebsiella* should also be considered.

## CONCLUSIONS AND FUTURE PERSPECTIVES

Overall, we have highlighted that there is a significant gap in the accurate identification and reporting of microorganisms associated with recall of contaminated non-food consumer products. We must change the accuracy of recall reporting information to avoid significantly under-reporting the presence of potentially pathogenic and known AMR organisms in non-food products. In this respect, the non-food consumer product industry and the microbiological analyses required of them, lag behind those of the food and clinical microbiology sectors. Going forward, it is paramount that microbial contaminants in non-food products are accurately identified and reported to isolate problematic organisms and sources of contamination, improve quality control and preservation system design, identify and characterise AMR organisms and ultimately protect consumers.

## AUTHOR INFORMATION

RW conceived and initiated the metadata analysis of non-sterile food products. EC-O updated the analysis and conceived the comparison to food product recalls. EM and TP secured funding support. EC-O and EM drafted the initial manuscript, and all authors contributed to further review, analysis and writing.
